# Innate Lymphoid Cells in Tumor Immunity

**DOI:** 10.3390/biomedicines4010007

**Published:** 2016-02-25

**Authors:** Jasper J. P. van Beek, Anne W. J. Martens, Ghaith Bakdash, I. Jolanda M. de Vries

**Affiliations:** 1 Department of Tumor Immunology, Radboud University Medical Center, Radboud Institute for Molecular Life Sciences, 6525 GA Nijmegen, The Netherlands; Jasper.vanBeek@radboudumc.nl (J.J.P.v.B.); Anne.Martens@student.uva.nl (A.W.J.M.); Ghaith.Bakdash@radboudumc.nl (G.B.); 2 Department of Medical Oncology, Radboud University Medical Center, Radboud Institute for Molecular Life Sciences, 6525 GA Nijmegen, The Netherlands

**Keywords:** innate lymphoid cells (ILCs), ILC1s, ILC2s, ILC3s, lymphoid tissue inducer (LTi) cells, cancer, cancer immunotherapy

## Abstract

Innate lymphoid cells (ILCs) are a group of immune cells of the lymphoid lineage that do not possess antigen specificity. The group includes natural killer (NK) cells, lymphoid tissue inducer (LTi) cells and the recently identified ILC1s, ILC2s and ILC3s. Although the role of NK cells in the context of cancer has been well established, the involvement of other ILC subsets in cancer progression and resistance is just emerging. Here, we review the literature on the role of the different ILC subsets in tumor immunity and discuss its implications for cancer treatment and monitoring.

## 1. Introduction

The immune system is specialized in the detection and eradication of tumor cells that have developed following a failure of intrinsic tumor-suppression mechanisms. In the initial phase of the anti-tumor immune response, tissue-resident innate immune cells, such as macrophages, are stimulated to produce pro-inflammatory cytokines and chemokines. This results in the attraction of other innate cells, including natural killer (NK) cells that can directly lyse transformed cells. In a later phase, dendritic cells (DCs) take up tumor antigens released by dying tumor cells and present those in the lymph node to naïve T cells, resulting in a wave of antigen-specific cytotoxic T lymphocytes (CTLs) and helper T cells that further aid tumor destruction. Increased understanding of all players involved in tumor immunity is important for the development of new, and improvement of current, cancer therapies. Recently, the family of innate cells to which NK cells belong, has been expanded to include newly identified members. These innate lymphoid cells (ILCs) are characterized by a classic lymphoid cell morphology, but lack lineage-specific markers and somatically rearranged antigen receptors. Based on their cytokine profiles and set of expressed transcription factors, they have been divided into three distinct subclasses: group 1 ILCs, group 2 ILCs and group 3 ILCs [1].

Group 1 ILCs are characterized by their ability to secrete interferon (IFN)-γ and include NK cells and ILC1s [1]. Upon activation, NK cells release perforin and granzyme molecules, allowing them to lyse targets like virus-infected or transformed cells [2]. In addition, they secrete pro-inflammatory cytokines. As their anti-tumor effector functions have been extensively reviewed elsewhere [3], they are out the scope of this review. ILC1s highly express the transcription factor T-bet and can be subdivided based on CD127 expression. Where CD127^low^ ILC1s are mostly present in the intraepithelial layer [4], CD127^high^ ILC1s are found in the lamina propria [5]. ILC1s respond to IL-12, IL-15 and IL-18, upon which they can secrete IFN-γ and tumor necrosis factor (TNF)-α [4,5,6]. Similar to T helper 1 (Th1) cells, ILC1s mediate immunity against intracellular bacteria and parasites [6,7]. However, they can play a pathogenic role in chronic inflammation, as numbers of ILC1s are elevated in the intestine of Crohn’s disease patients [4,5].

ILC2s make up group 2 ILCs and are dependent on transcription factor GATA-binding protein 3 for their development and maintenance [8,9]. They can be stimulated by IL-33, IL-25 or thymic stroma lymphopoietin (TSLP), alarmins secreted by epithelial cells upon cellular stress and tissue damage [10,11]. ILC2s release predominantly IL-5 and IL-13, but have also been reported to secrete IL-4, IL-9 and amphiregulin [12,13,14,15]. They are involved in tissue repair [15], anti-helminth immunity [12,13,16] and allergic inflammation [10,17,18]. ILC2s are found in lung, skin and gut, but small numbers of circulating ILC2s can also be detected in blood [10].

Group 3 ILCs, which include lymphoid tissue inducer (LTi) cells and ILC3s, are characterized by the expression of transcription factor RAR-related orphan receptor (ROR)γt [19,20,21]. These cells can be stimulated by IL-1β and IL-23 and mainly produce IL-17 and/or IL-22 [22,23,24]. Whereas LTi cells promote the formation of tertiary lymphoid structures [25], ILC3s are involved in antibacterial immunity, tissue homeostasis and repair and chronic inflammation [22,26,27,28]. ILC3s can be subdivided based on expression of certain natural cytotoxicity receptors (NCRs): NKp46 in mice and NKp44 in humans [29]. Engagement of NKp44 on human NCR^+^ ILC3s has been associated with a more pro-inflammatory cytokine response, including the release of TNF-α, as opposed to the release of IL-22 which is induced upon stimulation with cytokines like IL-23 [30]. NCR^−^ ILC3s are mainly found in healthy skin [31], while NCR^+^ ILC3s are the predominant ILC subset in healthy intestine and tonsil [29,32].

Based on transcriptional activity, cytokine profiles and effector functions, it is clear that ILCs strongly resemble the different helper T cell subsets: ILC1s and Th1 cells, ILC2s and Th2 cells and ILC3s and Th17 cells. In a similar way, NK cells can be viewed as the innate counterpart of CTLs. The role of helper T cells, CTLs and NK cells in tumor resistance and progression has been largely established, but data about ILCs are limited. However, these often overlooked immune cells may play a critical role in tumor immunity. As they do not require antigen sensing for their activation, ILCs respond more rapidly than adaptive T cells. While NK cells circulate in the blood, the other ILCs reside in mucosa and mucosal-associated lymphoid tissues. These ILCs are therefore possibly among the first to respond to, or induce formation of, tissue-resident tumors. Their effects are mostly related to cytokines released upon activation and thus vary among the different subsets. Depending on the secreted cytokines and the specific tumor microenvironment, ILCs may either aid anti-tumor immune responses or promote tumor formation and growth. Here, we summarize each subset in the context of tumor immunology.

## 2. ILC1s in Tumor Immunity

The main effector function of activated ILC1s is the production of IFN-γ, while lower levels of TNF-α might also be produced. Although there is currently no direct evidence linking ILC1s with tumor immunity, the effect of their secreted cytokines has been extensively investigated.

IFN-γ acts directly on tumor cells to upregulate expression of major histocompatibility complex (MHC) class I, thereby increasing anti-tumor immune responses [33]. Furthermore, IFN-γ can inhibit tumor cell proliferation [34] and angiogenesis [35], while promoting apoptosis [36]. In addition to its direct anti-tumorigenic effects, IFN-γ also enhances activity of cytotoxic effector cells, including CTLs, NK cells and macrophages [37,38,39]. Moreover, it is an important cytokine for the polarization of CD4^+^ T cells into Th1 cells, that in turn contribute to anti-tumor CTL and macrophage responses [40,41]. However, it should be noted that IFN-γ may have pro-tumorigenic effects in other settings, where it is implicated to actually enhance proliferation and to mediate tumor resistance to CTL- and NK-mediated killing [42].

TNF-α has been shown to mediate anti-tumor immunity as well. It mediates recruitment and stimulation of macrophages and DCs, resulting in strong anti-tumor responses [43,44]. Furthermore, *in vivo* models demonstrate that TNF-α signaling is required for CTL generation and that TNF-α secreted by CTLs directly induces tumor rejection [45,46]. However, dysregulated TNF-α signaling can also promote tumor formation and growth. On a cellular level, TNF-α induces growth [47], angiogenesis [48] and migration [49]. It mediates epithelial-mesenchymal transition [50], leading to a loss of cell-cell contact and thereby enhanced migration, which is a hallmark of metastasis. Mouse models of colitis-associated colorectal cancer (CRC) and skin cancer show that blocking of TNF-α can prevent tumor formation [51,52].

**Figure 1:**
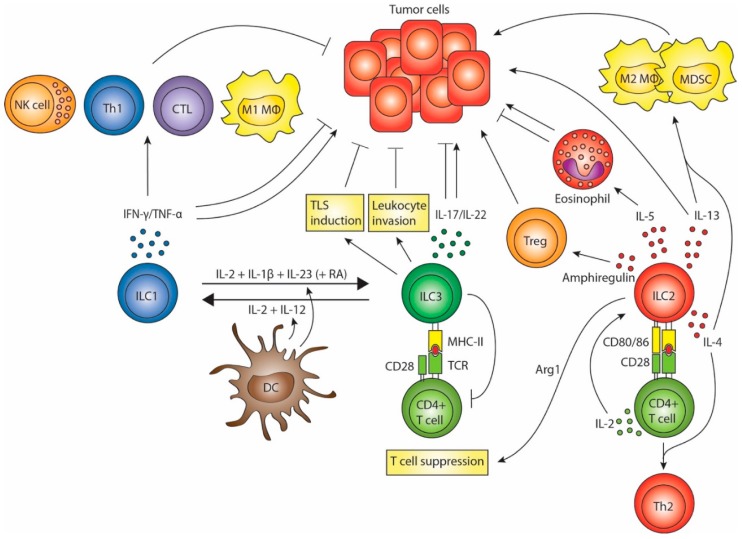
ILC (Innate lymphoid cell) interactions in tumor immunity. ILC1s secrete IFN-γ and TNF-α, which can have anti- or pro-tumorigenic effects. ILC3s may promote tumor formation and progression by secreting IL-17 and IL-22 and possibly by suppressing T cell responses. Conversely, ILC3s might promote anti-tumor responses by enhancing leukocyte invasion, promoting tertiary lymphoid structure induction and through the anti-tumor effects of IL-17 and IL-22. ILC2s may contribute to tumor progression either directly through the tumorigenic effects of IL-13, or indirectly by stimulating M2 macrophages and myeloid-derived suppressor cells through IL-13 and IL-4. ILC2-associated eosinophilia could inhibit metastasis formation, but may also exert pro-tumorigenic effects. Production of amphiregulin and arginase-1 suggests that ILC2s may inhibit T cell responses either directly or via stimulation of regulatory T cells. Finally, ILC2s can skew CD4^+^ T cells to a Th2 phenotype in an indirect (via secretion of type 2 cytokines) or direct manner (via activation of naïve CD4^+^ T cells). Whether these effects of ILC2s on T cells hamper anti-tumor responses is not known. ILCs are functionally plastic. ILC1s can differentiate into ILC3s and vice versa, a process which is mediated by DCs.

Both IFN-γ and TNF-α can thus play a dual role in tumor immunity. It seems likely that ILC1s are able to promote anti-tumor immunity (Figure 1), though their role might be hampered in cancer patients. This was shown in acute myeloid leukemia patients, where circulating ILCs display a reduced capacity to produce IFN-γ and TNF-α [53]. The anti-tumor potential of ILC1s has been corroborated by a recent study describing ILC1-like cells expressing NK1.1, CD49a and CD103. Unlike CD127^+^ ILC1s, these cells express granzyme B and TRAIL and can efficiently lyse tumor cells, which was crucial for immunosurveillance in a mammary tumor model [54]. It remains to be established whether this cell type represents a new entry in the group 1 ILCs and whether an equivalent exists in humans. Thus, further research is required to elucidate the role and interactions of ILC1s in cancer.

## 3. ILC2s in Tumor Immunity

Where type 1 responses favor tumor immune surveillance, type 2 responses are generally associated with an environment promoting tumor formation and progression. The central role of IL-33 in inducing tumor-promoting type 2 responses has recently gained attention. Administration of IL-33 in a murine model of breast cancer resulted in increased tumor growth and development of metastases, which was correlated with increased intratumoral numbers of IL-13-producing ILCs, IL-13 receptor α 1-expressing myeloid-derived suppressor cells (MDSCs) and regulatory T cells (Tregs) [55]. IL-33 stimulates ILC2s to secrete large amounts of IL-13, which has been shown to activate tumor-promoting MDSCs and their production of anti-inflammatory transforming growth factor-β (TGF-β) [56]. In addition, IL-13 can polarize macrophages towards a pro-tumorigenic M2 phenotype [57]. Although direct evidence is currently lacking, it hence seems likely that ILC2s contribute to tumor immune evasion via IL-13-mediated stimulation of tumor-associated myeloid cells.

ILC2-derived IL-13 might also induce *de novo* malignant transformation. Patients suffering from liver cirrhosis have elevated serum levels of IL-33 [58]. In a mouse model of hepatic fibrosis, the effects of IL-33 were shown to be related to activation and proliferation of liver-resident ILC2s. Their production of IL-13 activated hepatic stellate cells, resulting in hepatic fibrosis [59]. Liver fibrosis is linked to an increased risk of developing hepatocellular cancer [60]. Since ILC2s appear to play a role in the development of fibrosis, it is possible that these cells are involved in the transition from fibrosis to hepatocellular cancer as well. More direct evidence for a role of ILC2s in carcinogenesis exists for cholangiocarcinoma. Administration of IL-33 in a mouse model of biliary atresia induced increased numbers of ILC2s that secreted IL-13, ultimately resulting in cholangiocyte hyperplasia [61].

ILC2s may have an anti-tumor effect as well. Besides secreting IL-13, activated ILC2s produce high amounts of IL-5. This cytokine acts on eosinophils, which can release a storm of various cytokines, cytotoxic granules, cationic proteins and other factors. IL-5-producing ILC2s were shown to inhibit the formation of lung metastases in mice via recruitment and activation of eosinophils [62]. Depletion of IL-5 in this model resulted in an increase of metastases. Nevertheless, eosinophils have also been implicated to promote tumor growth and are associated with a poor prognosis in several cancer types [63,64].

Furthermore, ILC2s express molecules that are associated with T cell suppression. An increased frequency of circulating ILC2s can be detected in the blood of gastric cancer patients [65]. This is accompanied by an increase in IL-33, IL-25, IL-5 and IL-4, but paradoxically a decrease of IL-13. Interestingly, mRNA levels of iNOS and arginase-1 (Arg1) are increased as well, which may indicate an increase in MDSCs and M2 macrophages. Arg1 is an enzyme that metabolizes l-arginine into urea and l-ornithine. When T cells are depleted of l-arginine, it results in expression of defective T cell receptors and a halt of their cell cycle progression [66,67]. Increased Arg1 activity in the tumor microenvironment by myeloid cells can therefore lead to tumor immune evasion [68]. Arg1 production in murine macrophages is induced by IL-4 and IL-13 [69,70], cytokines that can be produced by ILC2s. Moreover, ILC2s themselves constitutively express Arg1 under both homeostatic conditions and during helminth infections [71]. Hence, it is possible that ILC2s inhibit T cell responses in the tumor microenvironment via their Arg1 activity and upregulation of Arg1 in myeloid cells. ILC2s might also induce immune suppression via secretion of amphiregulin [72]. Amphiregulin enhances Treg activity *in vivo* and can thereby inhibit anti-tumor immune responses induced by DC vaccination [73].

Finally, ILC2s can alter adaptive immune responses by skewing naïve CD4^+^ T cells to a Th2 phenotype. In T cells, IL-4 induces STAT6-mediated expression of GATA-binding protein 3, the master transcription factor of Th2 cells [74]. Cytokines like IL-4 act as a polarizing signal (signal 3) during T cell priming. For naïve T cells to become effector cells however, they also require signal 1 (T cell receptor binding of antigen-presenting MHC) and signal 2 (co-stimulation). By providing a type 2 cytokine milieu, ILC2s can therefore steer differentiation of activated T cells [75]. In addition, ILC2s may also directly activate naïve T cells, by acting as antigen-presenting cells. ILC2s can express both MHC class II and co-stimulatory molecules, including OX40L, ICOS, CD80 and CD86 [75,76,77,78]. Culture of CD4^+^ T cells with antigen-pulsed ILC2s *in vitro* leads to T cell differentiation into Th2 cells and a blockade of Th1 differentiation [77,78]. ILC2-T cell crosstalk also leads to proliferation and increased cytokine secretion of ILC2s, which is mediated by T cell-derived IL-2 [77,78]. Moreover, depletion of ILC2 *in vivo* causes diminished Th2-mediated responses [18,77]. In one such model, ILC2-derived IL-13 was critical for migration of DCs from the lung to draining lymph nodes where they were able to activate and prime naïve T cells [18]. Thus, ILC2-mediated Th2 skewing can occur both locally and at a distance. Whether ILC2-induced Th2 skewing hampers adaptive anti-tumor responses remains to be established.

Collectively, these data mainly point towards an immunosuppressive role of ILC2s in tumor immunity (Figure 1) and they therefore seem an interesting target for cancer immunotherapies.

## 4. ILC3s in Tumor Immunity

The pathogenic role of ILC3s has almost exclusively been studied in the gut. This group of innate cells was recently shown to be involved in inflammatory bowel disease (IBD). IBD, which includes ulcerative colitis and Crohn’s disease, is characterized by chronic inflammation of the intestine. Critical in immune-mediated chronic inflammation is the IL-23/IL-17 cytokine axis [79]; IL-23, which is upregulated in active disease, mediates the release of the pro-inflammatory cytokine IL-17 from Th17 cells. IL-23 also acts on ILC3s to release IL-17 and this was essential for the development of bacteria-driven colitis in *Rag^−/−^* and *T-bet^−/−^.Rag2^−/−^* mice [26,80]. In support of these findings, the inflamed intestine of Crohn’s disease patients show increased expression of *IL17A* and *IL17F* among CD3^−^ cells [81]. Interestingly, tissue-infiltrating neutrophils were identified as the main source of IL-23 in IBD [82]. Furthermore, IL-17 signaling can synergize with TNF-α to induce secretion of neutrophil-attracting chemokines by intestinal epithelial cells [80]. Hence, a positive feedback loop might be formed between IL-23-producing neutrophils and IL-17-producing Th17 cells and ILC3s. Long-standing inflammation can promote tumor formation and as such, patients suffering from IBD are at an increased risk of developing CRC [83]. This raises the question whether ILC3s are also involved in the formation of malignancies.

There is ample evidence that IL-23, the upstream mediator of ILC3s, is involved in carcinogenesis. Human colon tumors have elevated levels of IL-23 compared to healthy tissue [84]. Furthermore, mice deficient for IL-23 are resistant to tumor formation induced by chemical carcinogenesis and the growth of transplanted tumor cell lines was inhibited in mice deficient for the IL-23 receptor [84]. Blockade of the IL-23 receptor also inhibits bacterial-induced colonic tumor formation [85]. Transgenic expression of IL-23 in wild-type mice even results in adenoma formation independent of carcinogens [86]. In this particular model, IL-17-producing ILC3s were essential for the adenoma formation. In human CRC, IL-17 promotes angiogenesis via vascular endothelial growth factor (VEGF) production by tumor cells and is correlated with a poor prognosis [87]. The association between IL-17 and angiogenesis has also been established in other human cancers, including breast [88], lung [89], pancreatic [90], hepatocellular [91] and gastric cancer [92]. In addition to its angiogenic effects, IL-17 may also induce MDSCs and their recruitment at the tumor site, as demonstrated by experiments performed in tumor-bearing mice [93].

ILC3s may also release large amounts of IL-22 upon stimulation by IL-23. Under homeostatic conditions, IL-22 is inhibited by IL-22 binding protein (IL-22-BP), a soluble receptor secreted by immature DCs [94]. During cellular damage, IL-22BP is downregulated, allowing IL-22 to induce tissue repair. However, aberrant proliferation induced by IL-22 may promote tumor growth [95]. Moreover, in a colitis-associated colon cancer model, IL-22BP-deficient mice showed accelerated tumor development and an increase in quantity and size of tumors compared to wild type control mice, suggesting an important role for IL-22 in colitis-associated CRC [96]. In line with these results, IL-22-producing ILC3s were shown to promote tumor growth in a mouse model of bacteria-induced CRC, as depletion of IL-17^+^ IL-22^+^ ILC3s prevented the development of malignancies [97]. In this model, IL-17 only played a minor role in promoting tumor growth. Blocking of IL-17 merely reduced inflammation, while blocking of IL-22 led to a significant decrease in tumor burden. IL-22 produced by ILC3s may play an important role in human CRC as well, as both IL-22^+^ CD3^+^ and IL-22^+^ CD3^−^ cells can be detected within CRC tumors [97].

Similar to ILC2s, NCR^−^ ILC3s can present antigen on MHC class II to T cells. However, only splenic ILC3s are able to induce T cell responses [98]. In contrast, intestinal ILC3s lack co-stimulatory molecules and thus limit T cell responses [99]. Instead, these intestinal ILC3s induce T cell death via outcompeting the T cells for IL-2. This mechanism of T cell inhibition is important for maintaining intestinal tolerance: mice with ILC3s lacking MHC class II have increased intestinal inflammation due to T cell responses against commensal bacteria [99]. Whether these activities also contribute to tumor immune evasion in the gut is currently unknown.

In sharp contrast, ILC3-produced IL-17 and IL-22 may also promote tumor immunity in other settings. IL-17 was shown to boost tumor-antigen specific CTL responses in mice engrafted with hematopoietic tumors [100]. In addition, IL-17-deficient mice inoculated with a colon cancer cell line had increased tumor growth and metastasis compared to wild type control mice, which was correlated with decreased NK and tumor-specific T cell responses [101]. Furthermore, IL-22 was shown to reduce tumor growth in a breast cancer model [102]. In addition, although prolonged IL-22 production promotes tumor growth in a colitis-associated colon cancer model, it is suggested that IL-22 in the early phase of colitis actually protects against tumor formation [96].

A study by Carrega *et al.* showed more direct evidence that ILC3s may exhibit anti-tumorigenic effects [103]. The authors found that, while only low numbers of NCR^+^ ILC3s are present in healthy lung tissue, a higher frequency is present in non-small cell lung cancer. NCR^+^ ILC3s especially accumulated on the edge of intratumoral tertiary lymphoid structures (TLSs), which are associated with favorable prognosis. Moreover, NCR^+^ ILC3s were correlated with increased TLS density and less advanced tumor stage, suggesting that via induction of TLSs they promote tumor immunity. Importantly, the NCR^+^ ILC3s can recognize and interact with both tumor cells and tumor-associated fibroblasts via NKp44. This results in significant secretion of TNF-α and IL-8, but only low amounts of IL-22 and IL-2. NCR^+^ ILC3s in mice have also been linked to tumor suppression. Administration of IL-12 in a melanoma model led to a reduction of tumor growth, which was mediated by NCR^+^ ILC3s [104]. The mechanism behind this tumor suppression involved an upregulation of adhesion molecules in the tumor microenvironment, leading to enhanced leukocyte invasion. Group 3 ILCs were also crucial for the anti-tumor effects of a combination of chemotherapy and antibodies directed against a tumor antigen in a melanoma mouse model deficient for adaptive immunity [105]. In this model, CD90^+^ NK1.1^−^ ILCs expressing RORγt promoted macrophage infiltration, which was suggested to be the cause of the tumor growth arrest.

LTi cells are phenotypically very similar to ILC3s. These cells are crucial for the development of the lymphatic system in the embryonic state, but remain present during adult life and are implicated to help maintain lymphoid structures [23]. They can take part in innate immune responses, as LTi cells were critical for resolving an infection of *Citrobacter rodentium* in mice [106]. Their role in tumor immunity is not clear and research is hampered by the lack of a good definition of this cell type. However, one study linked LTi cells with tumor immune evasion [107]. This study showed that melanoma cells overexpressing chemokine CCL21 promotes attraction of Tregs, MDSCs and naïve T cells and induces lymphoid-like stroma, resulting in enhanced tumor growth. CCL21 overexpression in LTi-depleted mice did not enhance tumor growth, suggesting that LTi cells play a role in CCL21-dependent immunological tolerance.

Altogether, the ambiguous role of the ILC3s in tumor immunity shows similarities with that of its adaptive counterpart, the Th17 cell. Whether the effect of ILC3s is tumorigenic or anti-tumorigenic seems to depend on the specific subset, the timing of their responses and the specific tumor microenvironment (Figure 1). Especially in inflammation-associated cancers, prolonged ILC3-mediated responses seem to promote tumor progression.

## 5. ILC Plasticity and Crosstalk with DCs

Not only do ILCs act directly on tumor and tumor-associated cells, they also possess the ability to steer adaptive T cell responses. Another cell type that should not be neglected in ILC biology is the DC. DCs form a crucial link between the adaptive and innate immune system [108]. By acting as professional antigen-presenting cells, they initiate antigen-specific T and B cell responses and are therefore essential for induction of a long-lasting anti-tumor immune response. Besides, DCs also interact with innate and innate-like immune cells such as NK cells, natural killer T cells and γδ T cells and these interactions might be utilized to improve upon current anti-cancer immunotherapies [109]. Evidence is now accumulating that DCs interact with ILCs as well, especially in the gut.

NCR^+^ ILC3s form the main ILC population present in healthy intestine. In contrast, the inflamed intestine of Crohn’s disease patients mostly contains CD127^+^ ILC1s. Bernink *et al.* have shown that this discrepancy is due to ILC plasticity [32]. The differentiation of ILC3s into CD127^+^ ILC1s *in vitro* can be mediated by either CD14^+^ DCs or addition of IL-12 [5,32]. It is likely that this process occurs in a physiological setting as well, as greater numbers of CD14^+^ DCs are present in inflamed tissue of Crohn’s disease patients and they show increased expression of *IL12A* [32]. This plasticity is reversible, as CD127^+^ ILC1s differentiate into NCR^+^ ILC3s when stimulated with IL-2, IL-1β and IL-23 [32]. Retinoic acid further enhances the conversion. These findings indicate a role of DC-induced ILC plasticity in gut immunity and homeostasis. An infection in the gut may cause an influx of CD14^+^ inflammatory DCs that release IL-12, resulting in the conversion of ILC3s into ILC1s. These ILC1s subsequently amplify inflammatory type 1 immune responses and aid in clearing the infection. When the infection is resolved, CD14^+^ DCs leave and tissue-resident CD14^−^ DCs can convert CD127^+^ ILC1s back into NCR^+^ ILC3s via secretion of IL-23 and retinoic acid. Taken together, DCs can control the fate of ILCs by exposing them to a certain cytokine milieu (Figure 1).

In Crohn’s disease, ILCs take part in an inflammatory feedback loop. Loss of mucosal integrity and exposure to microbial gut flora leads to a state of chronic inflammation. On one hand, this causes prolonged activation of ILC3s, resulting in secretion of high levels of IL-17 that further aggravate the inflammatory response [81]. On the other hand, it results in a persistent increase in IFN-γ-producing ILC1s that too enhance inflammatory responses and may therefore contribute to the disease [5]. With this in mind, Bernink *et al.* proposed that forcing a switch of IFN-γ-producing ILC1s to IL-22-producing ILC3s holds therapeutic effect in Crohn’s disease [32]. Consequently, this might also prevent the development of inflammation-associated tumors in those patients. One can speculate that in certain cancers, an imbalance between ILC subsets exists as well. Contrary to the chronic inflammatory status in Crohn’s disease, most tumor microenvironments dampen immune responses and thereby evade immune detection. Hence in this setting, an increase in IFN-γ-producing ILC1s might be beneficial.

*In vitro* and *in vivo* experiments have shown that colonic DCs are an important source of IL-23, the main activator of ILC3s and LTi cells [80,110,111]. However, ILCs might also activate DCs. In the murine gut, ILC3s were identified as the main producers of GM-CSF, which they release upon stimulation by macrophage-derived IL-1β [28]. GM-CSF is required for optimal differentiation and function of tissue-resident macrophages and DCs. Consequently, mice deficient for GM-CSF have reduced numbers and activity of macrophages and DCs [28]. This indicates that crosstalk between DCs and ILCs is bidirectional.

## 6. ILCs in Cancer Treatment

Classic ways to treat cancer include surgery, radiation and chemotherapy. In recent years, much progression has been made in what now is the fourth pillar of cancer therapy: immunotherapy. Cancer is now seen as a disease arising from inadequate resolving of neoplastic cells by the immune system. Tumor cells do not only evolve ways to evade the immune system themselves, via up- and downregulation of membrane molecules and secretion of soluble factors, but often create and depend on a mesh of other cells that help them in suppressing the immune system. Characterization of this tumor microenvironment has therefore become a useful tool for monitoring disease progression and predicting clinical outcome. Since different ILC subsets can exert either pro- or anti-tumorigenic effects, their detection in patient blood and/or biopsies might contribute to a better assessment of disease status. There are however some drawbacks to implementing ILC detection in disease monitoring. The presence of ILCs in peripheral blood is very low. They make up only 0.01% to 0.1% of all circulating lymphocytes and most of these ILCs are ILC2s [112]. Still, changes in these percentages may be detected in certain cancer types. It was reported that in acute myeloid leukemia, numbers of ILCs are decreased and show a relative increase of ILC1s and decrease of NCR^+^ ILC3s [53]. More research is needed to determine if changes in ILC populations have a prognostic value. However, since higher numbers of ILCs reside in mucosal tissues, detection of ILCs in patient biopsies seems preferable over detecting them in blood. Yet obtaining multiple biopsies during the course of the disease puts a big burden on patients. Another hurdle to overcome is the amount of markers that are needed for ILC identification, which can be a limitation in particular for immunohistochemistry. Moreover, multiple different names and markers have been used in different studies for characterization of ILCs [1]. Advances in this field will therefore require the development of standardized protocols for ILC detection.

Besides their potential use as a prognostic marker, ILCs also pose as interesting targets for immunotherapy. Adoptive transfer of ILC subsets that exert anti-tumorigenic effects, or depletion or blocking of ILC subsets that promote tumor induction or progression can be two possible therapeutic strategies. The plasticity of ILCs could also be exploited to tip the balance between certain ILC subsets. In settings were ILC3s contribute to tumor development and progression, their conversion into IFN-γ-secreting ILC1s might be a method to improve tumor rejection. Cytokine injections could be one way to promote ILC polarization. However, administration of cytokines can have severe side effects, as with IL-2 immunotherapy is shown [113]. Another method could be DC vaccination, considering that DCs can induce phenotype switching of ILCs and mediate ILC activation. For this purpose, the injected DCs should be engineered in such a way that they secrete factors that mediate the generation and activation of the ILC subset of interest. Blocking factors that polarize ILCs in a disease-promoting subset should also be considered. Retinoic acid blocking in certain cancers might, among other effects, inhibit conversion of ILC1s into pathogenic ILC3s. Interestingly, blocking of the retinoic acid receptor in a mouse model of melanoma increases efficacy of DC vaccination [114]. This is caused by a reduction in Tregs and an increase in IL-12 production by DCs. Whether ILCs play a role in this model has not been examined. Still, it might be worthwhile to investigate whether therapies targeting ILCs can improve current treatment modalities.

One of the most significant breakthroughs in tumor immunotherapy is the use of the so-called checkpoint inhibitors, antibodies directed against immunomodulatory molecules, such as CTLA-4 or PD-1 [115]. Tumor and tumor-associated cells often overexpress ligands for immunomodulatory receptors, thereby holding back effective T cell responses. Checkpoint inhibitor-based therapy aims at removing these breaks. There is rational for combining this with strategies targeting ILCs. The use of checkpoint inhibitors brings the immune system in a more activated state, thus making it more susceptible to the effects of cytokines released by ILCs. Moreover, the potential role of certain ILC subsets to promote leukocyte invasion and lymphoid structure formation or maintenance could be utilized to enhance access of activated effector cells to the tumor site. This potential application of ILCs should therefore warrant further investigation. Whether ILCs themselves are also directly affected by checkpoint inhibitors is not known. However, it has been reported that NK cells of multiple myeloma patients can express PD-1 and that anti-PD-1 antibodies enhance the effector functions of these NK cells [116].

## 7. Concluding Remarks

Since the discovery of tissue-resident ILCs, it has become clear that these cells play an important role in mucosal immunity and are involved in immune-mediated diseases such as IBD. Much less is currently known about their involvement in cancer. Limited data suggests that these ILCs can promote tumorigenesis, mediate tumor maintenance or contribute to anti-tumor immune responses, depending on the specific ILC subset and the microenvironment. ILCs show plasticity in their phenotype and can be polarized by DCs in a cytokine-dependent manner. This plasticity can possibly be exploited in future cancer therapies. However, more clinical research is needed to define the effects of ILCs as well as their usefulness as prognostic markers or therapeutic targets in cancer treatment.
